# Determination of HER2 phosphorylation at tyrosine 1221/1222 improves prediction of poor survival for breast cancer patients with hormone receptor-positive tumors

**DOI:** 10.1186/bcr2230

**Published:** 2009-02-24

**Authors:** Thomas Frogne, Anne-Vibeke Laenkholm, Maria B Lyng, Katrine Lütken Henriksen, Anne E Lykkesfeldt

**Affiliations:** 1 Department of Tumor Endocrinology, Institute of Cancer Biology, Danish Cancer Society, Strandboulevarden 49, DK-2100 Copenhagen, Denmark; 2 Department of Pathology, Odense University Hospital, Winsloewparken 15, 5000 Odense C, Denmark; 3 Medical Biotechnology Center, University of Southern Denmark, J.B. Winsloewsvej 25.3, 5000 Odense C, Denmark

## Abstract

**Introduction:**

High expression of total HER2 protein confers poor prognosis for breast cancer patients. HER2 is a member of the HER family consisting of four receptors, HER1 to HER4. HER receptor activity is regulated by a variety of mechanisms, and phosphorylation of the C-terminal part of the HER receptors is a marker for active signaling. The importance of phosphorylation and thereby activation of the HER1 to HER4 receptors, however, has not been investigated concomitantly in breast tumors. In the present study we examined the importance of active HER signaling in breast tumor biopsies and paired metastases, by evaluating the expression of phosphorylated HER1, HER2, HER3, Erk, Akt and the total level of HER4 and HER2.

**Methods:**

Immunohistochemical analysis was performed on 268 primary breast tumors and 30 paired metastatic lesions from postmenopausal women with hormone receptor-positive breast tumors, who had received adjuvant tamoxifen therapy. The observed protein expression levels were analyzed for co-expression, for correlation to clinicopathological parameters and for prognostic value in relation to disease-free survival and overall survival. Lastly, the difference between protein levels in primary tumors versus metastasis was evaluated.

**Results:**

In the primary tumors, 8%, 18%, 14% and 15% of cases were scored positive for total HER2, pHER1, pHER2 and pHER3 expression, respectively. HER4 was expressed with strong intensity in 68% and at moderate intensity in 29% of cases. The activated forms of Akt and Erk were quite uniformly expressed in the categories; negative, moderate or strong. In univariate analysis, expression of total HER2, pHER1, pHER2 and pHER3 was significantly associated with poor disease-free survival. Strong HER4 expression was associated with prolonged disease-free as well as with overall survival. Expression of pAkt and pErk was not correlated with survival. In multivariate analysis, pHER2 expression was clearly an independent marker for poor disease-free survival and overall survival when tested against tumor size, tumor grade, nodal status and HER2. Lastly, comparison of HER receptor expression in metastatic versus primary tumors showed a significant increase in expression of pHER1 and pHER3 in the metastases.

**Conclusions:**

In hormone receptor-positive breast cancer, determination of pHER2 yields additional prognostic information about poor prognosis compared with the current clinical standard for measuring HER2.

## Introduction

The epidermal growth factor receptor family constitutes four members: HER1 (EGFR/ErbB1), HER2 (ErbB2), HER3 (ErbB3) and HER4 (ErbB4). The four receptors are activated by binding of numerous ligands, which leads to heterodimerization or homodimerization and subsequent phosphorylation of specific tyrosine residues in the intracellular region. This activation by phosphorylation results in regulation of a variety of cellular processes including cell proliferation and survival. Much evidence suggests that the kinases Akt and Erk mediate a substantial part of the HER signaling [1].

In breast cancer, excellent work has been done to investigate the significance of HER signaling for prognosis, both by characterization of the HER dimers [2] as well as of the ligands [3]. The most studied receptors in breast cancer are HER1 and HER2; overexpression of HER2 generally occurs in about 20% of breast carcinomas, and is more frequent in estrogen receptor-negative than in estrogen receptor-positive cases [4]. HER1 and HER2 overexpression is generally associated with more aggressive tumors and poor prognosis [2,5-7]. The importance of HER3 has been less investigated, but from *in vitro *studies it is becoming clear that HER3 is involved in tumor growth and also in resistance to both endocrine and HER-directed treatment [8-10]. Several recent clinical studies have found HER3 to correlate with adverse clinicopathological properties [2,11,12]. Association with better prognosis, however, has also been reported [13,14]. The role of HER4 in breast cancer has also been investigated – and in estrogen receptor-positive breast cancer cell lines, HER4 is often found to mediate growth inhibition and cell differentiation [15,16]. Analysis of tumor biopsies generally shows that expression of HER4 is associated with prolonged survival [2,6,14,17,18].

In the clinical setting today, only three protein biomarkers are used; estrogen receptor, progesterone receptor and HER2. The golden standard for determination of HER2 positivity is evaluation by immunohistochemistry (IHC); and for IHC equivocal cases, this is followed by fluorescence *in situ *hybridization (FISH) analysis for determination of gene amplification. Patients with HER2-positive tumors are candidates for treatment with the monoclonal anti-HER2 antibody trastuzumab. Numerous other approaches, however, are currently being evaluated in clinical trials for their ability to inhibit HER1 and/or HER2 or all four HER receptors [19]. It is therefore important to have good quality biomarkers to ensure that the patient is offered optimal treatment.

Our intention with the present study was to concomitantly evaluate the activation status of HER1, HER2 and HER3 and to determine their importance in hormone receptor-positive breast cancer. We hereby aimed to find new and superior biomarkers for detection of poor prognosis, and also to suggest new markers for future investigations regarding prediction of benefit from endocrine and/or anti-HER therapy. We therefore measured the levels of HER1, HER2 and HER3, which were phosphorylated at tyrosine sites described to be important for activation of the receptors [20], as well as the levels of the activated form of the downstream target kinases Erk and Akt. We were also interested in determining the amount of pHER4, but we were not able to find a commercially available anti-pHER4 antibody that was pHER4 specific and applicable in IHC analysis. Instead, we measured total HER4 expression using an antibody against the C-terminal region. Overall, we report the analysis of phosphorylated HER1, HER2, HER3, Erk, and Akt as well as total HER2 and HER4 expression levels in 268 hormone receptor-positive primary tumors and 30 corresponding metastases from postmenopausal women treated adjuvantly with tamoxifen.

## Materials and methods

### Patients

A total of 268 postmenopausal patients were used for this study, all of which underwent surgery for primary invasive breast cancer between 1989 and 2001 at Odense University Hospital. Patients were classified as high risk, according to age (<75 years) and/or tumor size (> 2 cm) and/or Bloom and Richardson grade (≥ 2) and/or positive nodal status. The high-risk patients received tamoxifen as the first-line adjuvant treatment and all, except a single case, were positive for either estrogen receptor and/or progesterone receptor. Owing to side effects, 25 patients were subsequently crossed over from tamoxifen to megace or letrozole. The only other criterion for entering this study was that both paraffin-embedded and frozen tumor tissue was present.

The standard clinicopathological parameters are presented in Table 1. HER2 positivity was determined according to current standardized guidelines for HER2 testing; that is, IHC followed by FISH analysis for cases scoring 2+. The analysis was performed with the HercepTest™ for IHC and with pharmDx™ for FISH (both from DAKO A/S, Glostrup, Denmark).

**Table 1:** Clinicopathological parameters

Parameter	*n*	%
World Health Organization diagnosis		
Invasive ductal	230	86
Invasive lobular	32	12
Ductal with ductal carcinoma *in situ*	2	1
Unknown	4	1
Total	268	
Tumor grade		
1	62	27
2	103	45
3	63	28
Total	228	
Tumor size		
<2 cm	85	32
2 to 5 cm	161	60
>5 cm	22	8
Total	268	
Nodal status		
0	22	8
1 to 3	149	56
≥4	97	36
Total	268	
Receptor status (estrogen receptor or progesterone receptor)		
Negative	1	0
Positive	267	100
Total	268	
HER2		
Negative	246	92
Positive	22	8
Total	268	
Recurrence		
No	179	67
Yes	89	33
Total	268	
Death		
No	146	54
Yes	122	46
Total	268	

Tumor grade was scored according to Bloom and Richardson. HER2 was analyzed by standard testing (that is, the HercepTest™), followed by fluorescence *in situ *hybridization analysis of the cases scoring 2+.

The total number of malignancy graded tumors was only 228; this was due to tumors of lobular subtype and tumors of rare subtypes, which were not graded. Table 2 presents the patient characteristics and treatment characteristics. It should also be mentioned that only a single patient experienced relapse while receiving tamoxifen.

**Table 2:** Patient and treatment characteristics

	Median (years)	Range (years)
Age	61	48 to 74
Time on tamoxifen	1.8	0.4 to 6.0
Time to recurrence	3.1	0.12 to 16.2
Time to death	5.9	0.26 to 15.7
Time of follow up	12.4	6.3 to 19.2

The analyses on the clinical material have been approved by the local ethics committee for Region South Denmark, S-VF-20040064.

### Tumor material and construction of tissue microarrays

Archival formalin-fixed and paraffin-embedded primary tumor tissue was used to generate tissue microarrays comprised of two 2 mm cores from each tumor, as previously described [21]. For positive controls, we included tissue cores of epidermis (pHER1), striated muscle (HER4) and of the MCF-7 cell line, which had been treated with the HER3/4 family ligand heregulin 1β (recombinant heregulin 1β, 396-HB/CF; R&D Systems Europe Ltd, Abingdon, UK; pHER2, pHER3, HER4). Cores on each tissue miroarray were therefore known to express a high amount of the six proteins evaluated in this study. Negative controls were obtained by omitting the primary antibody.

Tumor metastases were available from 30 patients and were mostly distant metastases; for example, from skin (six patients), bone (four patients), bone marrow (three patients), pleural effusions (three patients), soft tissue (four patients), peripheral lymph nodes (two patients) and visceral organs (eight patients).

### Immunohistochemistry

Serial sections of 5 μm thickness were cut from the tissue microarrays, dewaxed and rehydrated. To block endogenous peroxidase, the slides were incubated in 1.5% hydrogen peroxide in Tris-buffered saline for 10 minutes.

Antigen retrieval was performed by three methods. When staining for pHER1, pHER2, pAkt or pErk, heat-induced epitope retrieval was performed by microwave oven for 15 minutes in TEG buffer (10 mM Tris, 0.5 mM ethylene glycol tetraacetic acid, pH 9); for pHER3, the TEG buffer was replaced with 1 mM ethylenediamine tetraacetic acid at pH 8. Antigen retrieval for the HER4 staining was done by a 15-minute protease treatment.

Incubation with primary antibody for 60 minutes at room temperature was followed by detection of the primary antibody using the Advance™ HRP system (DAKO). The applied chromogen was 3,3'-diaminobenzidine and all stainings were performed in the Autostainer Plus Link Instrument (DAKO). After washing, the slides were counterstained with Meyer's hematoxylin for 30 seconds. The following antibodies were used: pHER1^1173 ^(dilution factor 1:200, catalog number 4407), pHER2^1221/1222 ^(dilution factor 1:200, catalog number 2243), pHER3^1289 ^(dilution factor 1:100, catalog number 4791), HER4^c-terminal ^(dilution factor 1:250, catalog number RB-9045), pErk^202/204 ^(dilution factor 1:1000, catalog number 4376) and pAkt^473 ^(dilution factor 1:200, catalog number 3787). The HER4 antibody was from Thermo Fisher Scientific (Fremont, CA, USA), whereas all other antibodies were from Cell Signaling Technology (Danvers, MA, USA), which in the datasheets for the antibodies against the individual HER receptor demonstrated no cross-reaction to the other HER receptors.

### Evaluation of immunohistochemistry data

Two different scoring methods were used in the present study. The markers with predominantly membrane staining (HER2, pHER1, pHER2 and pHER3) were scored according to the HercepTest™ guidelines. For markers with predominantly cytoplasmic staining (HER4, pAkt and pErk), the overall staining intensity was scored.

In the HercepTest™: 0 = no staining or membranous staining in <10% of invasive tumor cells; 1 = faint or barely perceptible membranous staining in >10% of invasive tumor cells; and 2 = moderate and 3 = strong complete membranous staining in >10% of invasive tumor cells, respectively.

The overall staining intensity (that is, staining of membrane, cytoplasm and/or nucleus) was scored if >10% of the tumor cells were positive. We used a scale from 0 to 2: 0 = negative, 1 = moderate and 2 = strong staining intensity.

Furthermore, nuclear reactivity of all stainings was scored as either negative or positive, again with a cutoff value of 10% positive tumor cells.

TF scored all primary tumors and A-VL scored all metastases as well as 25% of the primary tumors. The scores from the two observers were not different by McNemar's test, and the kappa statistic ranged from 0.8 to 1.0. The six investigated proteins were scored in two cores from each tumor in approximately 90% of cases, whereas the remaining cases were scored from a single core. In the rare cases where the two score values differed by 2, the highest score value was used [21].

### Statistical analysis

Spearman correlation coefficients and Fisher's exact test were used to determine significant associations between the expression levels of the investigated proteins as well as to the clinicopathological parameters. Survival curves were generated by Kaplan–Meier estimates followed by the log-rank test. The Cox proportional hazards model was used to evaluate the prognostic value of the markers both alone (univariate) and in combination (multivariate) with the current standard markers: tumor grade, tumor size, nodal status and HER2. Lastly, the Sign test was used to evaluate differences between the protein expression levels of primary and metastatic tumors. All tests were two sided and *P *< 0.05 was considered significant. SAS 9.1 software (SAS Institute Inc., Cary, NC, USA) was used for all analyses.

## Results

### Expression levels of phosphorylated HER1, HER2, HER3, Erk and Akt and total HER4

**Figure 1:**
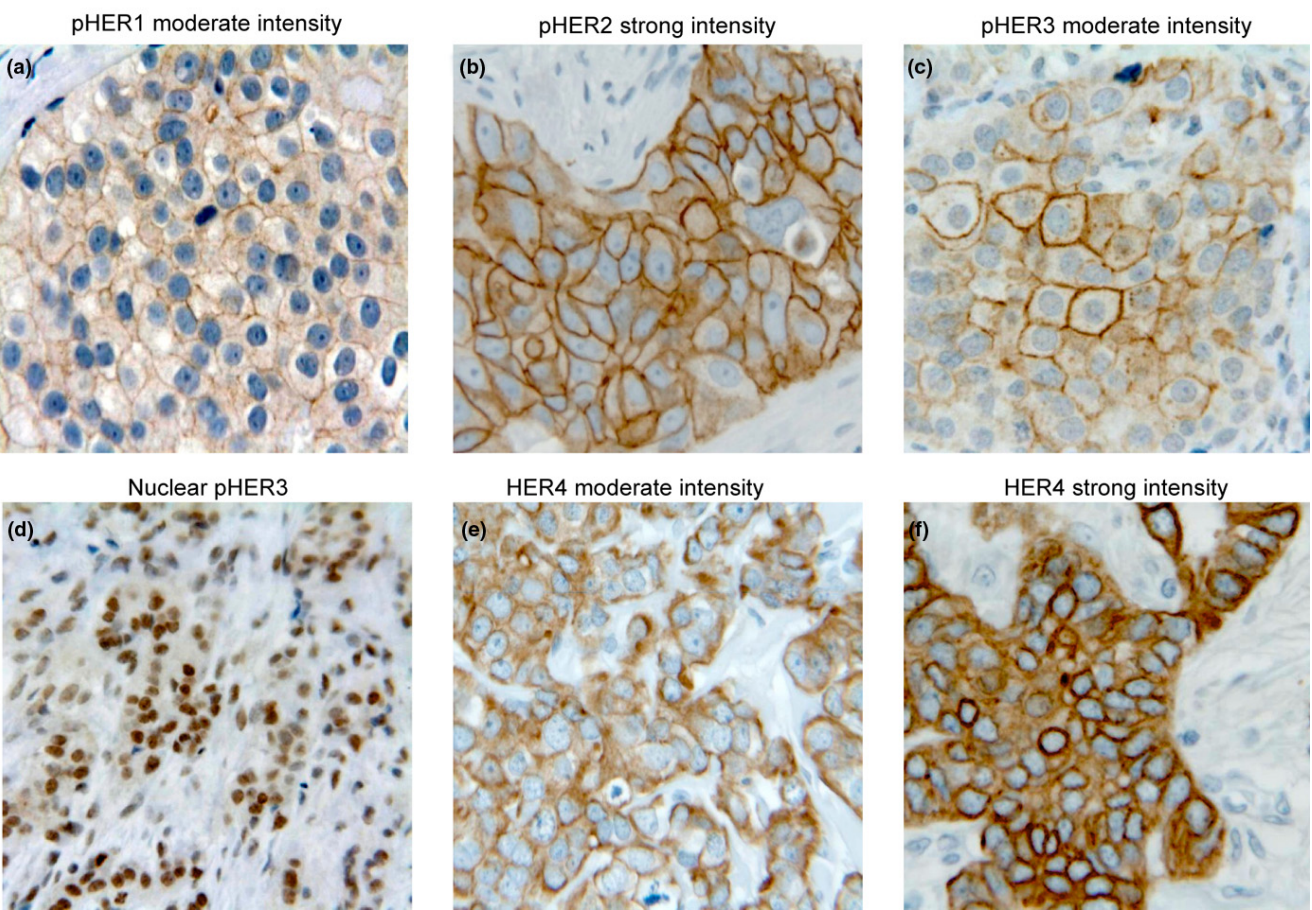
Immunohistochemical staining of primary breast tumors. **(a) **Moderate-intensity membranous pHER1. **(b) **Strong-intensity membranous pHER2. **(c) **Moderate-intensity membranous pHER3. **(d) **Nuclear pHER3. **(e) **Moderate HER4 intensity. **(f) **Strong HER4 intensity.

Membrane reactivity of pHER1, pHER2 and pHER3 was evaluated using the HercepTest™ score method, as described in Materials and methods. Expression of pHER1 was evaluated in 264 cases and 18% had a detectable membrane staining, of which the majority were weak (score 1) and a few showed moderate intensity (score 2). No tumors with strong (score 3) pHER1 membrane staining were observed. Expression of pHER2 was analyzed in 264 tumors and membrane staining with scores 1, 2 and 3 was recorded in 27%, 11% and 4% of cases, respectively. The moderate and strong pHER2 membrane reactivity was very often accompanied by cytoplasmic staining. We evaluated pHER3 expression in 261 cases and the strongest expression was found in the membrane and nucleus, but staining was also observed in the cytoplasm. Weak membrane staining was observed in 11% of cases, and only 3% and 1% were recorded with the scores 2 and 3, respectively. Interestingly, we also observed substantial nuclear pHER3 staining in 10% of the 261 cases. Expression of HER4 was evaluated in 259 cases and the staining was predominantly cytoplasmic, but most often in conjunction with a variable amount of membrane reactivity. We did not observe nuclear reactivity with this HER4 antibody. Owing to the considerable amount of cytoplasmic reaction, the HER4 staining was not scored by the HercepTest™ but by the overall intensity, grouped as either negative (score 0), moderate (score 1) or strong (score 2). The moderate intensity was observed in 29% of cases, 68% showed the strong intensity and only 3% were negative. Figure 1 shows representative pictures of the observed staining patterns of the four HER receptors.

The same overall intensity score was used to record the expression level of pErk (256 cases) and of pAkt (261 cases). Both the pErk and pAkt stainings were cytoplasmic and nuclear. The pErk expression was quite heterogeneous especially in the nucleus, whereas pAkt was more homogeneous and the same intensity was usually observed in both the cytoplasm and nucleus. We observed a fairly even distribution of cases in the three categories, with a few more pErk-negative tumors and slightly more tumors expressing a moderate level of pAkt. Based on these initial frequency data, we decided to assess pHER1 and pHER3 as either negative (score 0) or positive (scores 1, 2 and 3), to assess pHER2 as either negative (score 0), weak (score 1) or strong (scores 2 and 3), and to assess HER4 as either low (scores 0 and 1) or high (score 2). The pErk and pAkt scores were analyzed in the original categories.

### Correlations between protein expression levels

**Figure 2:**
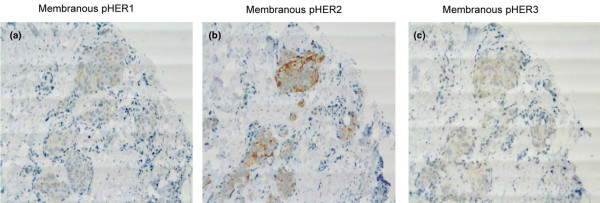
Immunohistochemical staining of primary breast tumor positive for activated forms of HER1, HER2 and HER3. Immunohistochemical staining of serial sections of a primary breast tumor showing that the same cells are positive for the activated forms of HER1, HER2 and HER3. **(a) **Membranous pHER1. **(b) **Membranous pHER2. **(c) **Membranous pHER3.

Table 3 presents the protein expression levels and their frequencies in relation to each other. Fisher's exact test was used to determine significant correlations between the investigated protein expression levels. We found strong positive correlation between membrane expression of HER2, pHER2 and pHER3. Briefly, 68% of the HER2-positive cases also showed strong pHER2 expression, while 28% of HER2-positive cases displayed weak pHER2 expression. Only a single patient therefore had a HER2-positive tumor without concurrent expression of pHER2. Conversely, only 39% of the strong-expressing pHER2 cases were found to be HER2-positive. Of the remaining HER2-negative and pHER2-positive cases (23 cases, 61%), 15 were scored 0 with the HercepTest™, six were scored +1, and two were scored +2 and without amplification. The HER2-negative cases with strong pHER2 expression were in 82% of cases expressed concomitant with pHER1 and/or pHER3. pHER2-positive cases that were scored HER2-positive expressed pHER1 and/or pHER3 in 74% of cases. The activated HER2 is therefore generally co-expressed with at least one other activated HER receptor that has the capacity to function as a dimerization partner. We used serial sections, and Figure 2 is a representative picture showing co-expression of all three phosphorylated receptors in the same tumor cells. Lastly, pHER2, but not HER2, was negatively correlated to HER4 expression.

**Table 3:** Correlations between protein expression levels

	HER2		pHER1		pHER2			pHER3		HER4		pErk		
	
	(-)	(+)	(-)	(+)	(-)	Weak	Strong	(-)	(+)	Low	High	(-)	Moderate	Strong
pHER1														
Negative	202	15												
Positive	40	7												
*P *value	0.083													
pHER2														
Negative	154	1	148	7										
Weak	65	6	55	15										
Strong	23	15	13	25										
*P *value	**<0.0001 **(+)		**<0.0001 **(+)											
pHER3														
Negative	211	10	199	21	144	61	14							
Positive	28	12	13	26	6	10	24							
*P *value	**<0.0001 **(+)		**<0.0001 **(+)		**<0.0001 **(+)									
HER4														
Low	75	10	69	16	49	16	19	71	13					
High	163	12	142	31	102	53	19	145	27					
*P *value	0.234		0.865		**0.021 **(-)			1.000						
pErk														
Negative	103	10	97	16	70	25	17	99	12	44	65			
Moderate	56	5	47	14	31	21	9	52	9	17	42			
Strong	75	7	65	15	46	24	11	64	17	22	60			
*P *value	1.000		0.328		0.495			0.146		0.073				
pAkt														
Negative	66	4	63	7	55	9	6	64	4	28	39	48	15	7
Moderate	99	7	88	17	58	30	17	87	17	34	71	40	22	37
Strong	74	11	64	21	40	30	15	66	18	21	62	23	22	37
*P *value	0.213		0.057		**0.002 **(+)			**0.021**(+)		0.102		**<0.0001 **(+)		

Fisher's exact test was used to obtain the *P *values. (+) and (-), positive and negative correlations. Bold data represent significant values.

Expression of the activated form of the intracellular kinases Akt and Erk was highly correlated, and pAkt was also associated with expression of pHER2 and pHER3 – and perhaps also with pHER1 (*P *= 0.057). No significant correlations between pErk and the HER receptors were observed.

### Associations between protein expression levels and standard clinicopathological parameters

Table 4 presents the classification of the observed protein expression levels in relation to the parameters of tumor grade, tumor size, nodal status, recurrence and death. From these distributions we used Fisher's exact test to analyze for potential significant correlations. Expression of HER2, pHER1 and pHER2 was found to correlate with recurrence. For HER2 and pHER2 we also found a positive correlation with high tumor grade, but interestingly only pHER2 was associated with the event of death. In contrast, we observed negative correlations between HER4 expression and grade, recurrence and death. Lastly, pAkt and pErk were not associated with the standard clinicopathological parameters.

**Table 4:** Protein expression levels in relation to clinicopathological properties

	Tumor grade			Tumor size			Nodal status			Recurrence		Death	
	
	1	2	3	<2 cm	2 to 5 cm	>5 cm	0	1 to 3	≥4	No	Yes	No	Yes
HER2													
Negative	61	96	49	78	149	19	22	137	87	169	77	137	109
Positive	1	7	14	7	12	3	0	12	10	10	12	9	13
*P *value	**0.0003 **(+)			0.506			0.335			**0.034 **(+)		0.190	
pHER1													
Negative	50	82	49	62	136	19	16	125	76	150	67	121	196
Positive	10	20	14	20	24	3	6	21	20	25	22	22	25
*P *value	0.731			0.188			0.180			**0.042 **(+)		0.333	
pHER2													
Negative	43	57	24	49	92	14	12	88	55	105	50	91	64
Weak	17	26	22	20	47	4	8	42	21	55	16	41	30
Strong	1	19	17	12	22	4	2	16	20	16	22	12	26
*P *value	**0.0001 **(+)			0.811			0.189			**0.001 **(+)		**0.009 **(+)	
pHER3													
Negative	50	88	50	69	134	18	20	123	78	153	68	122	99
Positive	10	15	13	12	25	3	2	21	17	22	18	20	20
*P *value	0.582			1.000			0.607			0.099		0.606	
HER4													
Low	10	32	27	24	49	12	7	41	37	38	47	36	49
High	51	67	35	56	109	10	13	105	57	133	42	104	71
*P *value	**0.004 **(-)			0.088			0.174			**<0.0001 **(-)		**0.012 **(-)	
pErk													
Negative	20	45	35	32	72	9	12	57	44	76	37	63	50
Moderate	13	25	11	18	40	3	4	33	24	42	19	32	29
Strong	22	32	16	27	45	10	5	50	27	52	30	42	40
*P *value	0.261			0.514			0.593			0.798		0.817	
pAkt													
Negative	10	29	20	20	46	4	8	33	29	48	22	42	28
Moderate	30	34	26	38	59	9	9	54	43	64	42	58	48
Strong	20	39	16	23	54	8	5	55	25	60	25	42	43
*P *value	0.103			0.588			0.195			0.287		0.419	

Fisher's exact test was used to obtain the *P *values. (+) and (-), positive and negative correlations. Bold data represent significant values.

### Kaplan–Meier plots for the HER receptors in relation to disease-free survival

**Figure 3:**
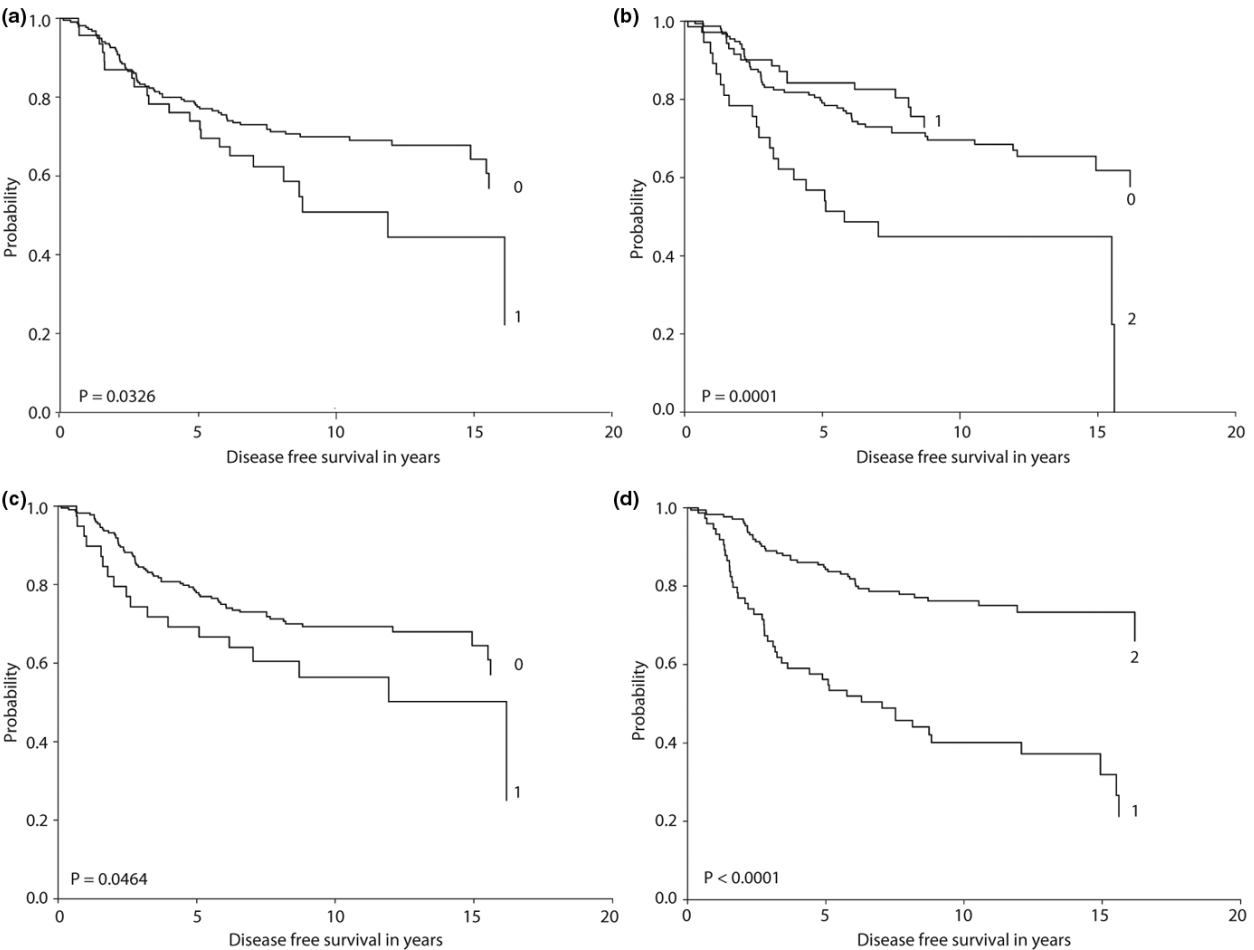
Kaplan–Meier curves showing disease-free survival in relation to protein expression levels. **(a)** pHER1: negative (0), positive (1). **(b) **pHER2: negative (0), weak (1), strong (2). **(c) **pHER3: negative (0), positive (1). **(d) **HER4: moderate (1), strong (2).

Figure 3 shows the Kaplan–Meier plots of pHER1, pHER2, pHER3 and HER4 expression levels in relation to disease-free survival. All four markers were found to be significantly associated with disease-free survival. Most of all, strong but not weak expression of pHER2 was highly associated with decreased disease-free survival. Likewise, although not as significant, expression of pHER1 and pHER3 was also found to correlate with reduced disease-free survival. In contrast, a high amount of HER4 was clearly associated with prolonged disease-free survival.

### Cox univariate and multivariate regression analysis of variables in relation to disease-free survival and overall survival

Based on the Kaplan–Meier plots we performed a Cox regression with the variables analyzed in the following categories: tumor grade (1 and 2 vs. 3), tumor size (<25 mm vs. ≥ 25 mm), nodal status (0 vs. ≥ 1), HER2 (negative vs. positive), pHER1 (negative vs. positive), pHER2 (negative and weak vs. strong), pHER3 (negative vs. positive), and HER4 (low vs. high). The results from the univariate Cox analysis are presented in Table 5; as expected, we found that high tumor grade, large tumor size and positive nodal status were significantly associated with poorer disease-free survival and overall survival. Also as anticipated, HER2 positivity was associated with poor disease-free survival; however, this positivity did not reach statistical significance in relation to overall survival (*P *= 0.06). For the other HER receptors, the Cox analysis confirmed the Kaplan–Meier plots with respect to disease-free survival, but interestingly we also found that strong pHER2 was highly associated with a reduction in overall survival. Likewise, a high amount of HER4 correlated with an increased period of overall survival. Lastly, we did not find expression of pErk or pAkt to be associated with survival in this patient series (data not shown).

**Table 5:** Cox univariate regression analyses of variables in relation to disease-free survival and overall survival

Variable	Disease-free survival		Overall survival	
	
	*P *value	Relative risk (95% CI)	*P *value	Relative risk (95% CI)
Tumor grade	**0.0201**	1.767 (1.093 to 2.86)	**0.0072**	1.785 (1.170 to 2.725)
Tumor size	**0.0007**	2.070 (1.357 to 3.160)	**0.0052**	1.662 (1.164 to 2.375)
Nodal status	**<0.0001**	3.193 (2.091 to 4.876)	**<0.0001**	2.205 (1.545 to 3.148)
HER2	**0.0037**	2.470 (1.342 to 4.546)	0.0595	1.739 (0.978 to 3.093)
pHER1	**0.0346**	1.683 (1.038 to 2.726)	0.2317	1.309 (0.842 to 2.033)
pHER2	**<0.0001**	2.699 (1.662 to 4.384)	**0.0002**	2.273 (1.470 to 3.513)
pHER3	**0.0489**	1.686 (1.002 to 2.835)	0.3926	1.233 (0.763 to 1.995)
HER4	**<0.0001**	0.361 (0.238 to 0.549)	**0.0111**	0.624 (0.433 to 0.898)

Variables were analyzed as follows: tumor grade (1 and 2 vs. 3), tumor size (<25 mm vs. ≥ 25 mm), nodal status (0 vs. ≥ 1), HER2 (negative vs. positive), pHER1 (negative vs. positive), pHER2 (negative and weak vs. strong), pHER3 (negative vs. positive), HER4 (low vs. high). 95% CI, 95% confidence interval. Bold data represent significant values.

For the multivariate Cox regression we initially included all variables in a single model, which showed that only positive nodal status and high HER4 expression were significant predictors of decreased and prolonged survival, respectively (data not shown). This result is in agreement with the strong correlations between HER2, pHER1, pHER2 and pHER3, and therefore this analysis does not give a truthful picture of the importance of the three phosphorylated HER receptors. We therefore tested pHER1, pHER2 or pHER3 alone in a model containing the current standard of clinical biomarker parameters (that is, tumor grade, tumor size, nodal status and HER2). The results in Table 6 show that strong pHER2 expression was a significant and independent predictor of decreased disease-free survival. Furthermore, strong pHER2 expression highly significantly predicted for reduced overall survival, in contrast to the current HER2 analysis.

**Table 6:** Cox multivariate regression analyses of selected variables in relation to disease-free survival and overall survival

Variable	Disease-free survival		Overall survival	
	*P *value	Relative risk (95% CI)	*P *value	Relative risk (95% CI)
Tumor grade	0.544	1.17 (0.71 to 1.92)	0.187	1.35 (0.87 to 2.09)
Tumor size	**0.018**	1.76 (1.10 to 2.80)	0.060	1.48 (0.98 to 2.21)
Nodal status	**<0.0001**	3.11 (1.90 to 5.11)	**0.0001**	2.29 (1.50 to 3.49)
HER2	0.097	1.84 (0.90 to 3.78)	0.574	1.22 (0.62 to 2.40)
pHER2	**0.009**	2.14 (1.21 to 3.78)	**0.005**	2.10 (1.25 to 3.52)

Variables were included in the following categories: tumor grade (1 and 2 vs. 3), tumor size (<25 mm vs. ≥ 25 mm), nodal status (0 vs. ≥ 1), HER2 (negative vs. positive), pHER2 (negative and weak vs. strong). 95% CI, 95% confidence interval. Bold data represent significant values.

In this multivariate model, expression of pHER1 and pHER3 did not have independent prognostic value (data not shown).

### Comparison of frequencies of protein expression levels between 30 primary tumors and their corresponding metastases

In addition to the primary tumor material, we also analyzed the protein expression levels of the six markers in 30 distant metastases from 30 patients within the original group. Table 7 presents the expression frequencies in the primary tumor and in the metastatic tumors. The final column in Table 7 presents the *P *values from a Sign test showing that membrane expression of pHER1 and pHER3 as well as nuclear expression of pHER3 were significantly increased in the metastases. No difference in expression was observed with respect to pHER2. The pHER2 level in the 30 primary tumors was already markedly increased, however, compared with the average pHER2 expression level of the original 264 primary tumors. Lastly, the HER4 expression appeared to decrease, but this change was not statistically significant.

**Table 7:** Frequencies of protein expression levels between primary and metastatic tumors

Protein	Primary tumors			Metastatic tumors			Sign test, *P *value
		
	0	1	2	0	1	2	
pHER1	25	--	5	11	--	19	**0.0003**
pHER2	17	5	7	14	10	6	0.6072
pHER3	23	--	5	16	--	13	**0.0034**
Nuclear pHER3	27	--	1	15	--	14	**0.0002**
HER4	--	13	17	--	19	10	0.1435
Cytoplasmic pErk	10	4	15	6	12	12	1.0000
Nuclear pErk	10	3	16	6	6	18	0.4545
Cytoplasmic pAkt	3	19	8	10	5	14	0.8036
Nuclear pAkt	16	8	8	10	7	12	0.0768

Category 0 = number of negative cases, category 1 = number of weak-expressing (pHER2), low-expressing (HER4) or moderate-expressing (pErk and pAkt) cases, and category 2 = number of positive (pHER1 and pHER3) or strong-expressing (pHER2, pErk and pAkt) cases. Expression of pHER1, pHER3 and nuclear pHER3 was analyzed as negative or positive, hence '--' in category 1. HER4 expression was evaluated as either low or high, hence '--' in category 0. The Sign test was used to examine for significant differences in protein expression levels between primary and metastatic tumors. Bold data represent significant values.

## Discussion

The present study investigated the prognostic value of the activated HER1, HER2 and HER3 receptors, the total HER4 expression and the activated form of the downstream kinases Akt and Erk. We addressed this issue in a series of postmenopausal patients presenting with primary hormone receptor-positive breast cancer, who had all received adjuvant tamoxifen therapy.

Total HER2 expression is an independent predictor of poor prognosis [2,7] and is also a clinical target for treatment [22]. In this study, 8% of the patients had HER2-positive tumors in accordance with this being a hormone receptor-positive population, and HER2 positivity was associated with shorter disease-free survival. Fourteen percent of cases were recorded with strong membrane expression of HER2 phosphorylated at tyrosine 1221/1222, however, and this HER2 phosphorylation was associated with high tumor grade and with shorter disease-free survival and overall survival. We speculate that the reason for this difference is due to the fact that moderate or even low levels of HER2 may be sufficient to elicit a potent mitogenic signal, upon activation by dimerization with ligand activated HER1 or HER3. This hypothesis is not new [23], and especially the importance of the HER ligands for activation of the HER receptors has been substantiated by several reports [3,24-26]. In agreement, we find that the hormone receptor-positive breast cancer cell line MCF-7, which we score HER2-negative, has weak to moderate levels of HER2 mRNA and protein [27], and addition of the HER3/HER4 ligand heregulin 1β clearly abrogated the inhibitory effect of antiestrogen treatment equally well in both wild-type and HER2/HER3 overexpressing MCF-7 cells – suggesting that activation of even a low level of HER2 via dimerization of ligand-activated HER3 may suffice to protect against antiestrogen therapy [27].

A recent study has compared HER2 mRNA expression in MCF-7 cells with mRNA expression in tumors classified as HER2-negative or HER2-positive, and the HER2 mRNA level in MCF-7 cells was lower than in most HER2-negative tumors [28]. This supports tumors classified as HER2-negative perhaps having sufficient HER2 protein to elicit signal transduction upon activation, and thereby may explain why also some patients with HER2-negative tumors respond to trastuzumab treatment [28].

It is noteworthy that 82% of cases with strong pHER2 staining without HER2 overexpression were scored positive for pHER1 and/or pHER3, clearly supporting that HER2 may be activated via dimerization with another HER family member. This is further supported by the finding that pHER2 was often expressed in the same tumor cells that were also positive for pHER1 and/or pHER3 (Figure 2). Our multivariate analysis clearly revealed that IHC evaluation of tyrosine 1221/1222 pHER2 was significantly better than the current HER2 tests with respect to select patients with both poor disease-free survival and overall survival. Other studies have also indicated that pHER2 expression may provide additional survival information [23,29,30]. In two of these studies, IHC detection of pTyr 1248 was measured and only a small fraction of the HER2-positive cases (12%) was positive for pHER2 [23,29]. In the study by Cicenas and colleagues [30], the same antibody against pTyr1248 was applied in a highly sensitive chemoluminescence-linked immune assay. In that study, pHER2 expression was found in both HER2-positive and HER2-negative tumors (68% and 27%, respectively). In concert with our finding, the multivariate analysis showed that pHER2 was a marker of poor prognosis independent of HER2. We have tested the antibody against pTyr 1248, but found only weak staining in our positive controls, indicating that this antibody may not be sensitive enough for IHC analysis as also suggested by Cicenas and colleagues [30]. We therefore selected the antibody against pTyr 1221/1222 directed against phosphotyrosines, which like pTyr 1248 is related to HER2 receptor activation [20]. Our data support that the pTyr1221/1222 antibody is a good antibody for IHC detection of activated HER2.

Future studies shall validate the clinical usefulness of the pTyr 1221/1222 antibody with respect to predict response to endocrine therapy and also in relation to predict response to therapy directed against the activated HER2 receptor. Our finding that activated HER2 is expressed also in HER2-negative tumors indicates that these tumors may utilize HER2 receptor signaling to promote growth, and thus may be potential responders to treatment targeting the activated HER2. Besides trastuzumab, which has been found to be beneficial also in a fraction of the patients with HER2-negative tumors [28], targeted therapy may involve the monoclonal antibody Pertuzumab, which targets HER2 receptor dimerization [31], or treatment with the tyrosine kinase inhibitor Lapatinib, which targets the kinase activity of both HER2 and HER1 [32].

In the present series, membrane expression of phosphorylated HER3 at tyrosine 1289 was found in 15% of cases and was associated with shorter disease-free survival. No previous work has investigated the importance of phosphorylated HER3 in primary breast cancers, but most studies of total HER3 expression have found HER3 to correlate with adverse clinicopathological properties [2,5,6,11,33,34]. Contrary results have also been observed, however; for example, inverse association with local recurrence [2] and association with longer survival [13,14]. In breast cancer cell lines, HER3 is a promoter of cell growth and is required for HER2-mediated proliferation [35]. More recently, HER3 activation has also been shown to be important for both tamoxifen-resistant and fulvestrant-resistant cell growth [10,36], and a significant role for HER3 in resistance towards HER-directed therapy is also evident [37,38]. In the present patient series, membrane expression of pHER3 did not add prognostic information to the current clinical parameters, hence substantiating that HER2 is the dominant HER receptor and that the function of HER3 is primarily to act as a co-activator of HER2. Moreover, nuclear expression of pHER3 was found in 10% of the patient tumors, but it was not significantly associated with the parameters investigated in the present series.

We observed membrane expression of HER1 phosphorylated at tyrosine 1173 in 18% of cases, and to the best of our knowledge only two other studies have measured pHER1 in breast cancer biopsies. These investigations were carried out on 225 and 154 cases of advanced breast cancer, and 7% and 36% of cases displayed membrane staining, respectively [39,40]. In agreement with these studies, our work showed that pHER1 was significantly correlated to shorter disease-free survival. Moreover, high total HER1 expression is also generally related to poor prognosis [2,5,6]. Overall, however, the literature on the prognostic value of total HER1 expression in breast cancer is not completely clear [41]. Our analyses show that membrane expression of pHER1 did not add prognostic value to the current clinical parameters, thus indicating that HER1, like HER3, also mainly acts as a co-activator of HER2.

In contrast to the predominantly membrane staining of total HER2, pHER1, pHER2 and pHER3, the HER4 staining was primarily cytoplasmic but also membranous. The overall intensity scoring method was therefore applied. HER4 expression was found in 97% of cases. The high percentage of positive cases is in concert with the data from Abd El-Rehim and coworkers, who found 80% HER4-positive cases in a series of more than 1,500 patients [2], and another study recorded 82% HER4-positive cases [18]. When only reactivity in the membrane is scored, however, the observed frequencies are much lower: 21% [17], 14% [5], and 12% [6]. In agreement, we found 15% of cases to express HER4 in the membrane. In this series, high HER4 expression was inversely associated with pHER2 and tumor grade, and had a positive effect on disease-free survival and overall survival. Furthermore, high HER4 expression independently predicted for longer disease-free survival and overall survival, compared with the currently used parameters. This result is much in line with earlier data from both protein and mRNA analyses, where HER4 predicted prolonged survival in multivariate analysis [6,14]. Moreover, others have found associations between high HER4 expression and longer disease-free survival and/or overall survival compared with low or negative cases [17,18].

We also looked at the activated levels of the HER downstream kinases Erk and Akt – we found that pAkt was correlated with pHER2, pHER3 and pErk, whereas pErk was not correlated with any of the HER receptors. Our data therefore suggest that pAkt is likely to be an important downstream mediator of HER2/HER3 signaling, which is very much in line with the current knowledge [8]. We did not find associations, however, between pAkt or pErk and clinicopathological parameters or survival. For pAkt this is in agreement with a study of 691 cases where the authors did not find an association with survival [42]. In contrast, others have observed pAkt expression in 54% of 93 patients analyzed and have shown that pAkt was an independent marker for disease-free survival [43]. Also, another study of 399 cases found pAkt to be associated with decreased overall survival in univariate analysis [44]. Few studies have been conducted for pErk, and also here conflicting results have been obtained. Using both immunoblotting and IHC, it has been shown by multivariate analysis that pErk was a marker for prolonged survival [45], while others have reported that pErk independently predicted for reduced survival [46,47]. In our patient cohort, there was a trend for pErk to be associated with expression of HER4 (*P *= 0.07) and this would indicate a preferable effect of pErk, but this was not evident in the Cox analysis. Moreover, the number of pErk and pAkt targets is currently sought to be around 160 and 70 proteins, respectively [48,49]. This fact is likely to, at least partly, explain the discrepancies between the studies.

The comparison of the expression levels of the six proteins in 30 distant metastases and their corresponding primary tumors disclosed a significant increase in expression of pHER1 and pHER3 in the metastases. This observation fits well with the poor prognosis of cases with primary tumors expressing the phosphorylated receptors and, worthy of note, it indicates that pHER1 and pHER3 may be important activators of HER2 in metastatic tumor cell growth. We did not find other reports investigating pHER1, pHER2 and pHER3 levels in this setting, but eight other studies have, in agreement with our finding, consistently found that the total HER2 expression, evaluated by IHC, is unaltered between the primary and metastatic lesions [50]. Analysis of activated HER1 and HER3 in distant metastasis may therefore be helpful in the clinical setting. Lastly, we observed a significant increase in the number of cases with nuclear expression of pHER3 in the metastasis. No data on nuclear HER3 expression exist in breast cancer, but a recent report showed that nuclear HER3 was absent in nonmalignant prostate tissue, whereas it was highly expressed in the cancerous prostate tissue and associated with increased tumor grade [51]. Studies with more statistical power should therefore investigate whether increased nuclear expression of HER3 in metastatic lesions may be associated with an adverse prognosis.

## Conclusions

The present study shows that strong expression of activated HER2 as well as activated HER1 and HER3 is associated with poor prognosis in this series of hormone receptor-positive breast cancer patients. We conclude that expression of HER2 phosphorylated at tyrosine 1221/1222 is likely to hold currently unassessed information on poor prognosis in hormone receptor-positive breast cancer.

## Abbreviations

FISH: fluorescence *in situ *hybridization; IHC: immunohistochemical; p: phosphorylated.

## Competing interests

The authors declare that they have no competing interests.

## Authors' contributions

A-VL, TF and AEL were responsible for the study design. A-VL collected the samples and performed all IHC analyses. TF and A-VL evaluated the IHC stainings. MBL provided the clinical patient data. KLH assisted with the IHC analysis and data interpretation. TF performed the statistical analysis, interpreted the data and drafted the manuscript. AEL critically revised the data interpretation and the manuscript, and obtained funding for the study. All authors revised the manuscript for important intellectual content.
